# Reciprocal repression between Fgf8 and miR-133 regulates cardiac induction through Bmp2 signaling

**DOI:** 10.1016/j.dib.2015.08.009

**Published:** 2015-08-24

**Authors:** Carmen Lopez-Sanchez, Diego Franco, Fernando Bonet, Virginio Garcia-Lopez, Amelia Aranega, Virginio Garcia-Martinez

**Affiliations:** a Human Anatomy and Embryology, Faculty of Medicine, University of Extremadura, 06006 Badajoz, Spain; b Cardiovascular Development Group, Department of Experimental Biology, University of Jaén, CU Las Lagunillas B3-362, 23071 Jaén, Spain; c Pharmacy Services, Minimally Invasive Surgery Centre, Cáceres, Spain

## Abstract

This data article contains complementary figures and results related to the research article entitled “Negative Fgf8-Bmp2 feed-back is controlled by miR-130 during early cardiac specification” [15], which reveals what specific role miR-130 plays during the cardiac induction process. This study evidenced miR-130 a putative microRNA that targets Erk1/2 (Mapk1) 3′UTR- as a necessary linkage in the control of Fgf8 signaling, mediated by Bmp2. Thus, miR-130 regulates a negative Fgf8-Bmp2 feed-back loop responsible to achieve early cardiac specification. A significant aspect supporting our conclusions is given by the expression pattern of miR-130 during early cardiac specification, as well as by those results obtained after the designed experimental procedures. The data presented here reveal that miR-133 is also expressed within the precardiac areas during early cardiogenesis, pattern which is comparable to that of FGFR1, receptor involved in the Fgf8/ERK signaling pathway. Interestingly, our miR-133 overexpression experiments resulted in a decrease of *Fgf8* expression, whereas we observed an increase of *Bmp2* and subsequently of cardiac specific markers *Nkx-2.5* and *Gata4*. Additionally, our loss-of-function experiments -through *Fgf8* siRNA electroporation- showed an increase of miR-133 expression. Finally, after our Bmp2 experiments, we observed that miR-133 is upstream-regulated by *Bmp2*. All those results suggest that miR-133 also constitutes a crucial linkage in the crosstalk between *Fgf8* and *Bmp2* signaling by regulating the Fgf8/ERK pathway during cardiac induction.

## Graphical abstract

**Figure:**
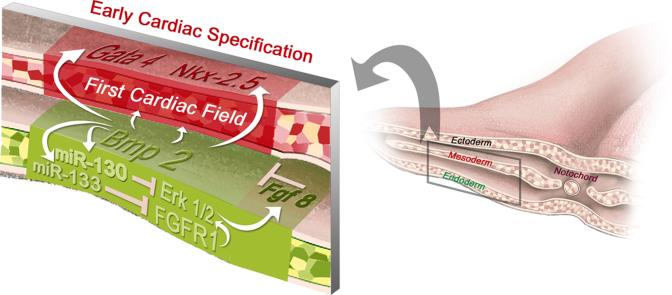

Specifications table

**Table:** 

Subject area	Biology
More specific subject area	Embryonic development
Type of data	Text file and figures
How data was acquired	TSSS20 Ovodyne Electroporator (Intracel), Nikon digital SIGHT DS-U1: bright and fluorescent light
Data format	Raw
Experimental factors	Electroporation, beads implantation, culture embryo
Experimental features	Whole-mount in situ hybridization, immunohistochemistry
Data source location	University of Extremadura, Badajoz, Spain
Data accessibility	The data are supplied with this article

Value of the data

• miR-133 modulates Fgf8 during cardiac induction.
• miR-133 is regulated by Bmp2.
• miR-133 exerts a regulatory role in Fgf8–Bmp2 signaling during early cardiac specification.

## 1 Data

Fgf8 constitutes a crucial factor involved in MAPK/ERK signaling pathway [8]. Also, FGF receptors have demonstrated to play a significant role during the early steps in the Fgf8/ERK signaling cascade [6,9]. Although there is no evidence about the role of FGF receptors during early cardiogenesis, there are previous studies where FGFR1 has been identified in the endoderm underlying the precardiac mesoderm [16,17]. However, its functional role has been preferentially involved in cell proliferation rather than in cell differentiation [19]. It has been reported that cardiomyocyte proliferation is suppressed after miR-133 overexpression [13], and that miR-133 is also involved in late stages of mouse cardiac development as well as in molecular mechanisms regulating adult cardiovascular diseases [1,2,7,12,18].

**Fig. 1:**
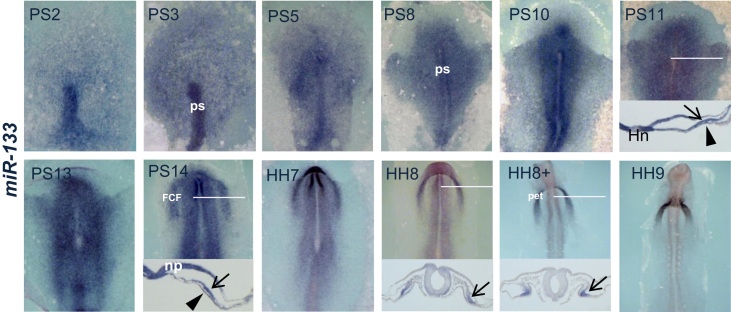
Whole-mount ISH analyses for *miR-133* during early chick development. Note the expression from the primitive streak (ps), through FCF, to the primitive endocardial tube level (pet). The white lines indicate the transverse section level, showing miR-133 expression in the precardiac mesoderm (arrow) and the underlying endoderm (arrowhead). Hensen׳s node: Hn. Neural plate: np.

To further analyze what mechanism regulates these signaling pathways, we will explore herein miR-133 expression and function. Although previous studies have provided data about miR-133 expression in chick embryo [5], it has only been analyzed in late stages of cardiac development. Thus, we have analyzed miR-133 expression from the primitive streak stages to the primitive cardiac tube formation (Fig. 1), expression which is already detectable at gastrula stages throughout the entire primitive streak, including the precardiac cells. Subsequently, its expression spreads laterally and concentrates into the first cardiac field (FCF) and the underlying endoderm, to finally express restrictively at the splachnic mesoderm of the primitive endocardial tube. This topographical location of miR-133 shows a similar distribution to that of *Fgfr1* [16,17]. Since FGFR1 has been proposed as a crucial factor involved in Fgf8/ERK signaling pathway in different experimental models [8,11], it is reasonable to consider a close relationship between miR-133 and *Fgfr1* during cardiogenesis.

**Fig. 2:**
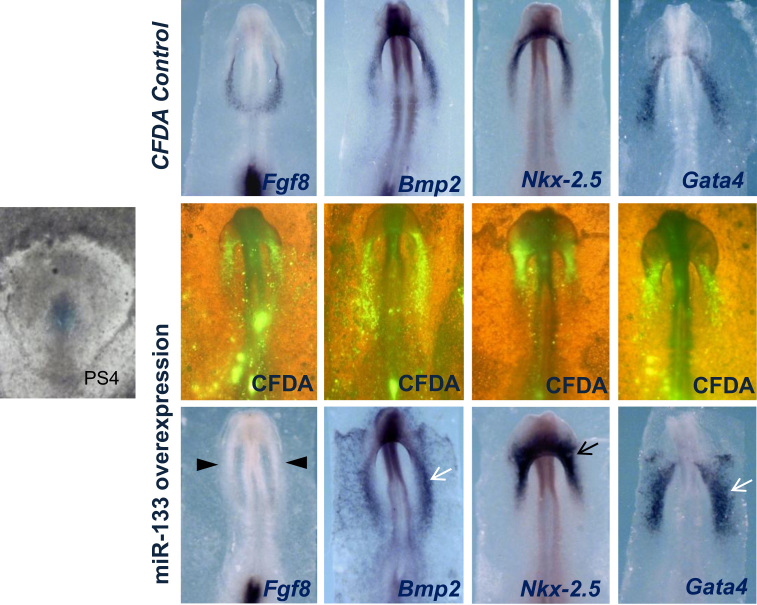
Effect of miR-133 gain-of-function on primitive endocardial tube specification. Embryos electroporated at the level of the primitive streak precardiac (left image) at stage PS4: Whole-mount ISH for *Fgf8, Bmp2*, *Nkx-2.5* and *Gata4*. Note the dramatic reduced *Fgf8* expression (arrowheads), whereas *Bmp2*, *Nkx-2.5* and *Gata4* are significantly increased (arrows), at the primitive endocardial tube level. Visualization of CFDA expression of embryos processed for ISH is shown in the immediate upper panel.

**Fig. 3:**
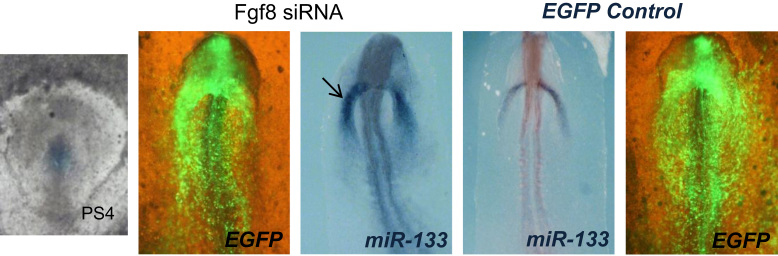
Effect of Fgf8 loss-of-function on primitive endocardial tube specification. Whole-mount ISH for miR-133. Embryos electroporated at the level of the primitive streak precardiac cells (left image), either with the control construct or *Fgf8* siRNA expressing construct. Note that at the primitive endocardial tube level miR-133 is markedly increased (arrow). EGFP expression of experimental embryos is shown in the immediate left panel.

**Fig. 4:**
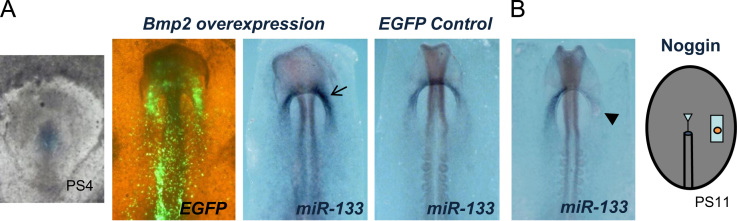
Effect of Bmp2 gain- and loss-of-function on primitive endocardial tube specification: whole-mount ISH for miR-133 *.* (A) Embryo electroporated at the level of the primitive streak precardiac at stage PS4 (left image), either with the control construct or with *Bmp2* expressing construct. Note the significant increase of miR-133 expression (arrow) at the primitive endocardial tube level. Visualization of EGFP expression of embryos processed for ISH is shown in the immediate left panel. (B) After noggin soaked bead application just inside FCF, at PS11 stage (right drawing), miR-133 is diminished at the level of the ipsilateral endocardial tube (arrowhead).

**Fig. 5:**
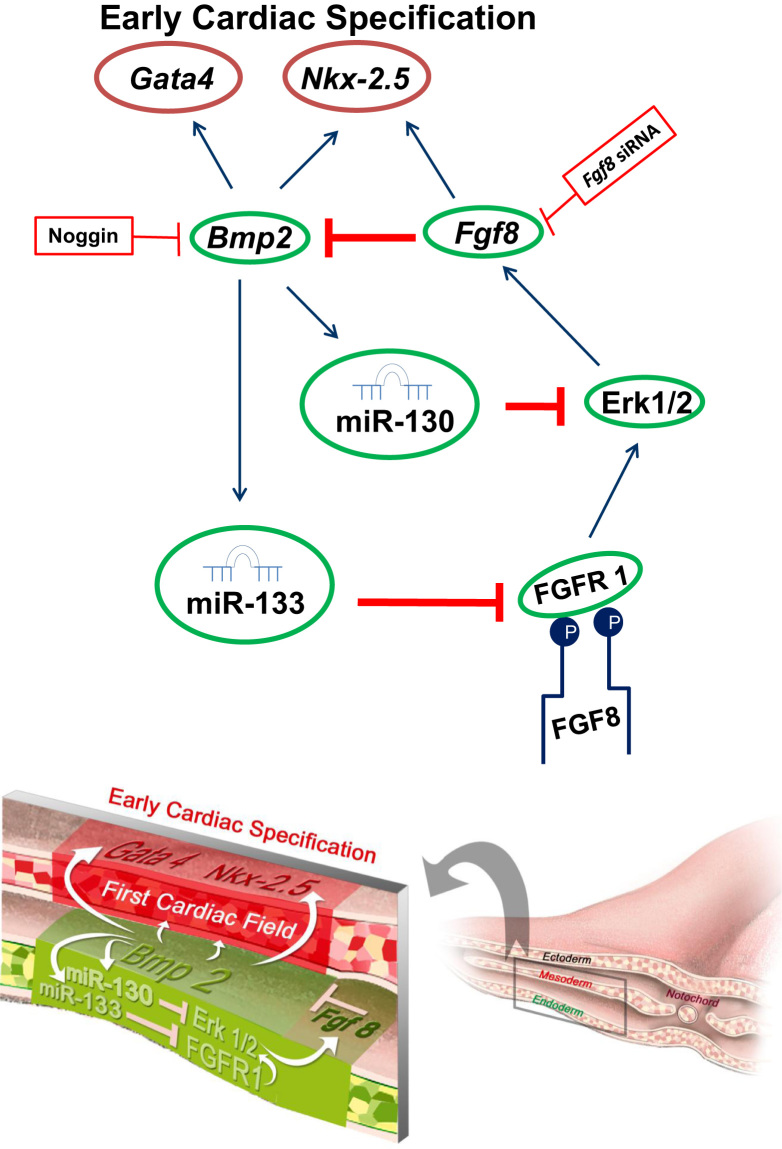
Proposed model for early cardiac specification. Our model indicates that the earliest cardiac markers -*Nkx-2.5* and *Gata4*- are induced by *Bmp2*, which is repressed by *Fgf8*. Moreover, *Bmp2* induces miR-130 and miR-133. Both repress Erk1/2 and FGFR1, respectively. Since *Fgf8* is controlled by these two factors -Erk1/2 and FGFR1, *Bmp2* modulates *Fgf8* expression. Thus, miR-130 and miR-133 act as necessary linkages in the control of Fgf8 signaling, mediated by Bmp2, establishing a negative feed-back loop responsible to achieve the initial cardiac specification.

To assess the potential involvement of miR-133 in Fgf8-Bmp2 cooperation during cardiogenesis, we analyzed the effects of miR-133 overexpression on the precardiac primitive streak cells, resulting in *Fgf8* inhibition and *Bmp2* increase. Subsequently, *Nkx-2.5* and *Gata4* increase as well (Fig. 2). Interestingly, our loss-of-function experiments through *Fgf8* siRNA electroporation showed an increased miR-133 expression (Fig. 3). It is also noteworthy that our *Bmp2* overexpression experiments induced miR-133 expression, whereas noggin soaked beads administration -specifically into FCF, showed a decrease of miR-133 expression in the ipsilateral endocardial tube (Fig. 4), suggesting that miR-133 is upstream-regulated by Bmp2. All the above results clearly indicate that a reciprocal repression between miR-133 and Fgf8 regulates cardiac induction through *Bmp2* signaling (Fig. 5), thus constituting complementary data to those obtained after our miR-130 analysis [15]. However, although miR-133 may be identified as a putative microRNA that targets FGFR1 3′UTR -site broadly conserved among vertebrates, its connection for Fgf8/ERK pathway in this process remains to be further established.

## 2 Experimental design, materials and methods

Fertilized eggs (Granja Santa Isabel, Córdoba, Spain) were incubated at 38 °C in forced-draft humidified incubators. Embryos were staged (PS stages: [14]; HH stages: [10]) and subjected to early chick (EC) embryo culture [3]. Two groups of embryos were selected for experiments:

### 2.1 Group 1. Embryo electroporation of precardiac primitive streak cells

Cultured embryos were injected and electroporated in precardiac primitive streak cells. For gain-of-function experiments, two different groups of embryos were electroporated with Bmp2 expressing construct (pIRES-Bmp2-EGFP) and pre-miR-133, respectively. For loss-of-function experiments, a group of embryos was electroporated with Fgf8 siRNA expressing construct (pSilencer-Fgf8). For control embryos, EGFP expressing construct (pCAGGs-EGFP), or CFDA, was electroporated. Embryos were additionally incubated for 14 to 16 h.

### 2.2 Group 2. Bead implantation

For loss-of-function experiments, beads were soaked in noggin (an antagonist of BMP signals) solution and implanted at the desired site. The embryos were additionally incubated for 6–8 h.

After the experimental procedures, the selected embryos were fixed overnight in 4% PFA, dehydrated in methanol and stored at −20 °C. Subsequently, they were processed for whole mount in situ hybridization: embryos were hydrated by incubation in graded methanol/PBT steps to a pure sterile PBT solution and processed for ISH following standard procedure [4] using antisense-*Nkx2.5, -Bmp2, -Fgf8,* and -*Gata4* labeled probes, respectively. A group of embryos was processed [5] for ISH with LNA-labelled microRNA probes (Exiqon) against miR-133.
